# Supplementary material for: Radiation tolerance of two-dimensional material-based devices for space applications
Source: Nat Commun. 2019 Mar 13;10:1202. doi: 10.1038/s41467-019-09219-5 (PMC6416293; doi:10.1038/s41467-019-09219-5)
Supplement: Supplementary file 1 — Supplementary Information [file 41467_2019_9219_MOESM1_ESM.pdf]

Supplementary Information: Radiation tolerance of two-dimensional material-based devices for space applications

Vogl et al.

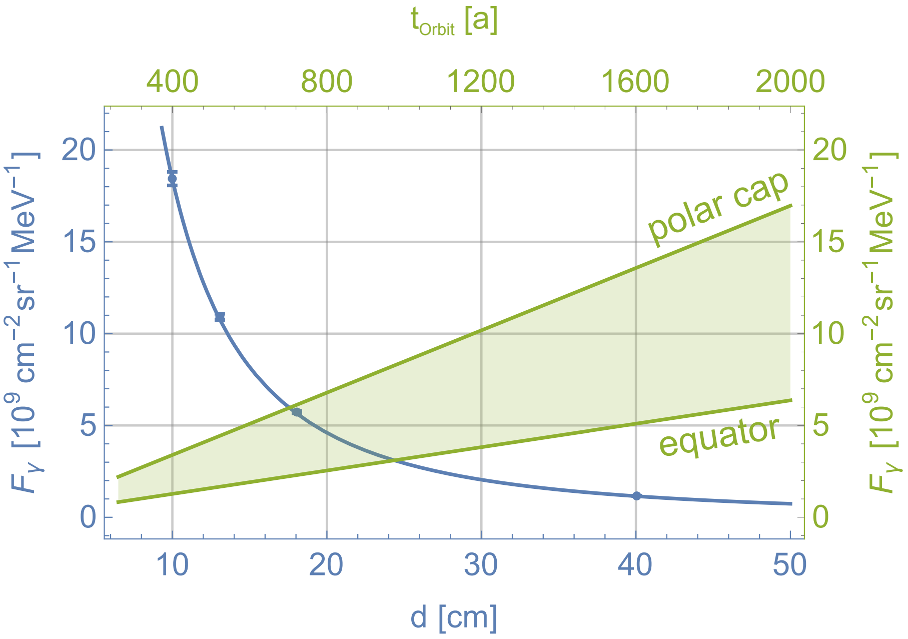

Supplementary Figure 1: Distance-dependent gamma-ray fluence. The fluence was calculated by taking the d^{-2} dependence of the photon flux into account. The data points mark the positions at which the samples were positioned during the first γ-ray test. The error bars denote the uncertainty resulting from the placement accuracy. We assume this to be ± 1 mm. The solid green lines show the integrated γ-ray flux as a function of orbit time for different geographical locations. Depending on orbital inclination, the real experienced value will be in between these lines (green shaded area). Due to a calculation error, (see main text) the simulated orbit times are 576 times higher than planned.

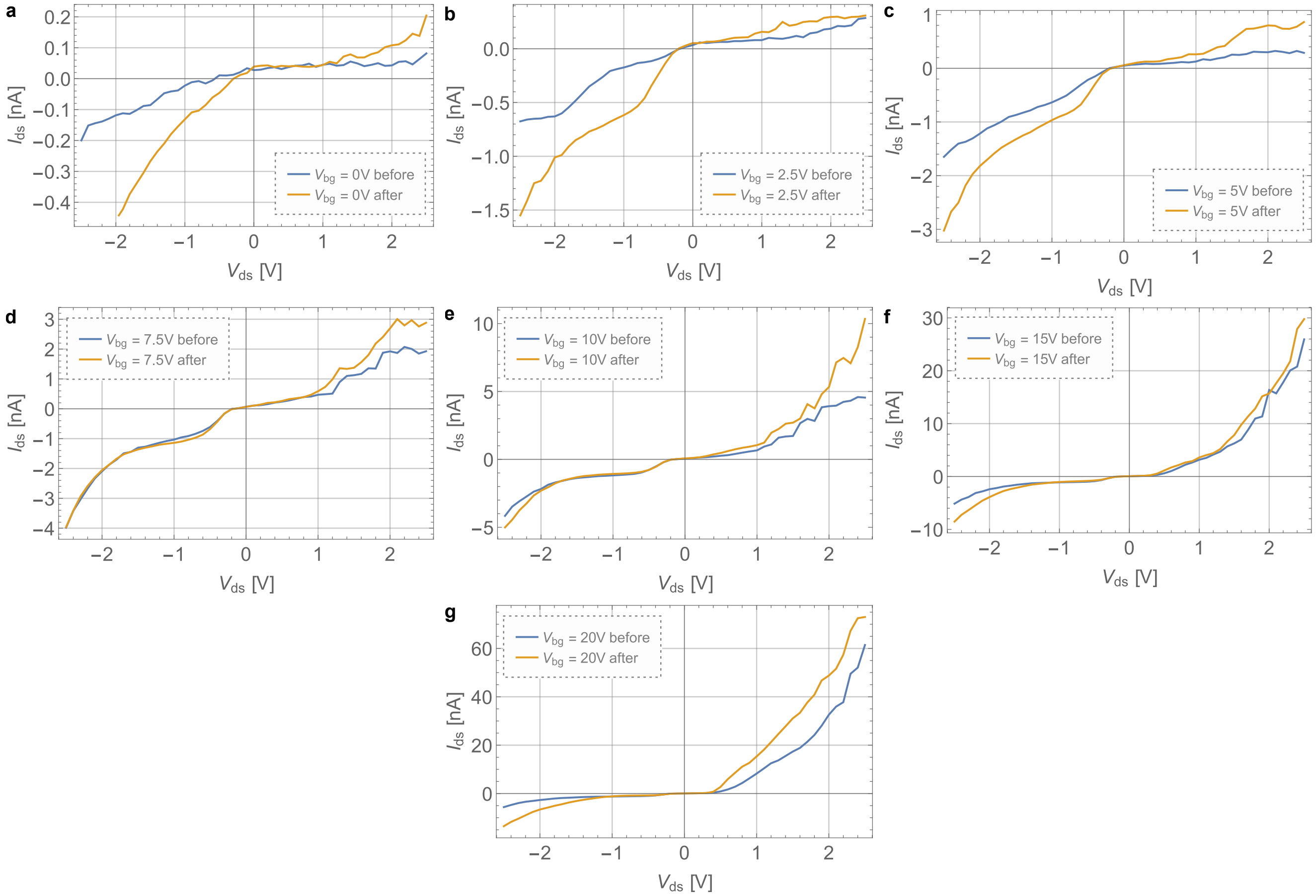

Supplementary Figure 2: Complete set of the I-V curves at different back gate voltages. **a-g** The variations in I-V characteristics before and after the γ-ray test were confirmed via time-dependent measurements to be most likely temporal variations, independet of any γ-ray exposure. These variations are likely due to surface adsorption, which changes the carrier mobility in such 2D materials (see main text). In addition, I-V characteristics in general are highly dependent on the Schottky or contact resistance which varies across different measurements. We confirmed this by measuring the same curve multiple times without irradiation at different days.

Supplementary Note 1: Averaging algorithm

The photoluminescence (PL) response typically varies across a monolayer, as can be seen from the confocal PL map in Supplementary Figure 3a. These variations are due to local contaminants on the crystal surface. We found that even by manually placing the excitation laser onto the same spot on a monolayer, the measured PL may vary. Thus, we averaged over the PL of the entire monolayer. For separating monolayer and multilayer/substrate, we first calculated the probability density function (PDF) of the entire PL map by kernel density estimation (KDE). The PDFs always show a bimodal distribution (see Supplementary Figure 3b). The first peak is attributed to substrate/multilayers and the second to the monolayer. Note that we have not truncated the PDF, resulting in a finite density below zero and above the maximal intensity I_{max} (in this case $I_{\mathrm{max}} = 690720\,\mathrm{a.u.}$). We select all data points with PL intensities between the local minimum between both peaks in the PDF and I_{max} (orange shaded area in Supplementary Figure 3b). The resulting selection mask is shown in Supplementary Figure 3c. We average over the selected data and the raw spectrum closest to the mean is assigned the average spectrum of the monolayer. For the lifetime such elaborate algorithm is not necessary. By mapping the lifetime of a crystal we found the average lifetime to be 319 ps with a standard deviation of 4 ps. Thus, the lifetime does not vary across a monolayer.

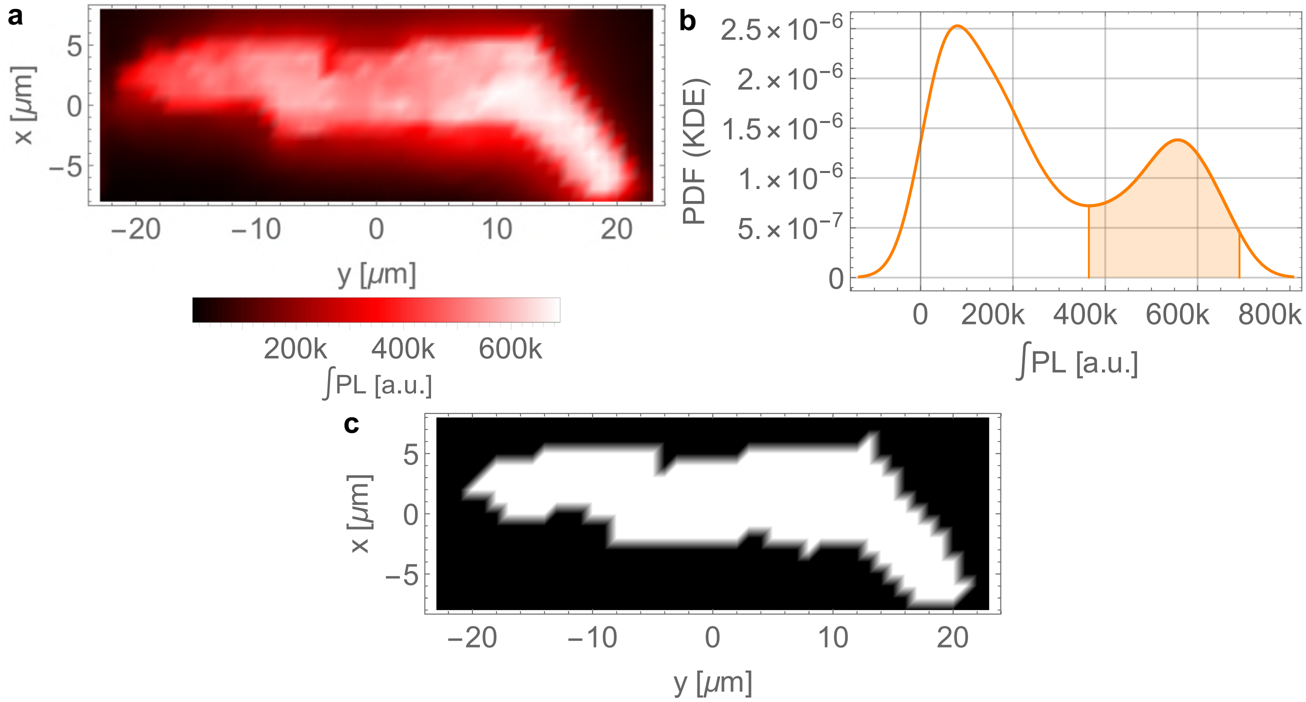

Supplementary Figure 3: Explanation of the averaging algorithm. **a** Confocal PL map of a monolayer. **b** PDF of the KDE. All scans show a bimodal distribution. The data in the orange shaded is used for averaging. **c** Map of the selection mask. All data falling into the white area is used for averaging.

Supplementary Note 2: Extended data gamma-ray tests

Note for all confocal PL maps: The black bars contain excluded data points at which the laser was re-focused during the scan. The data is shown in Supplementary Figures 4 through 8.

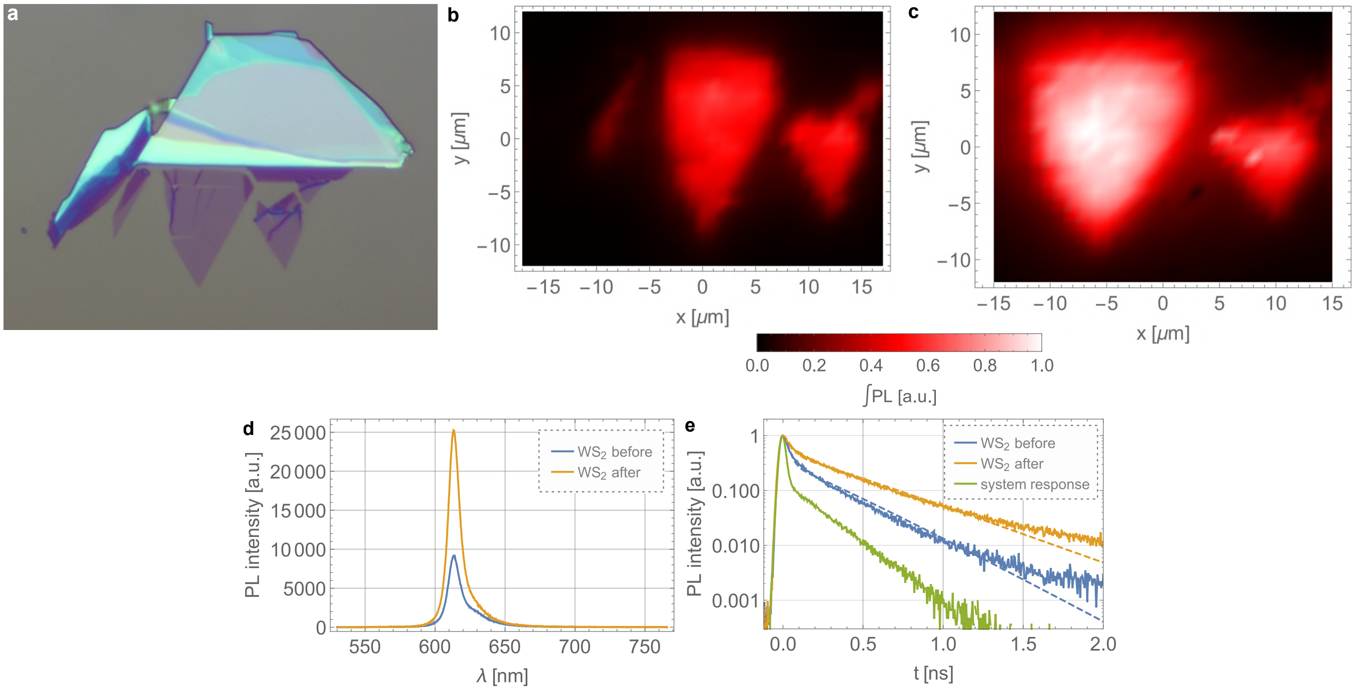

Supplementary Figure 4: Additional data set WS_2 after $F_\gamma = 10.89 \times 10^9\,\mathrm{cm^{-2}sr^{-1}MeV^{-1}}$. **a** Microscope image under 500× magnification. **b, c** Confocal PL map before and after irradiation. **d** The spectrum shows a PL increase of $\eta_{\mathrm{PL}} = 2.30$. **e** Carrier lifetime is increased from 272(2) to 417(3) ps ($\eta_\tau = 1.53$).

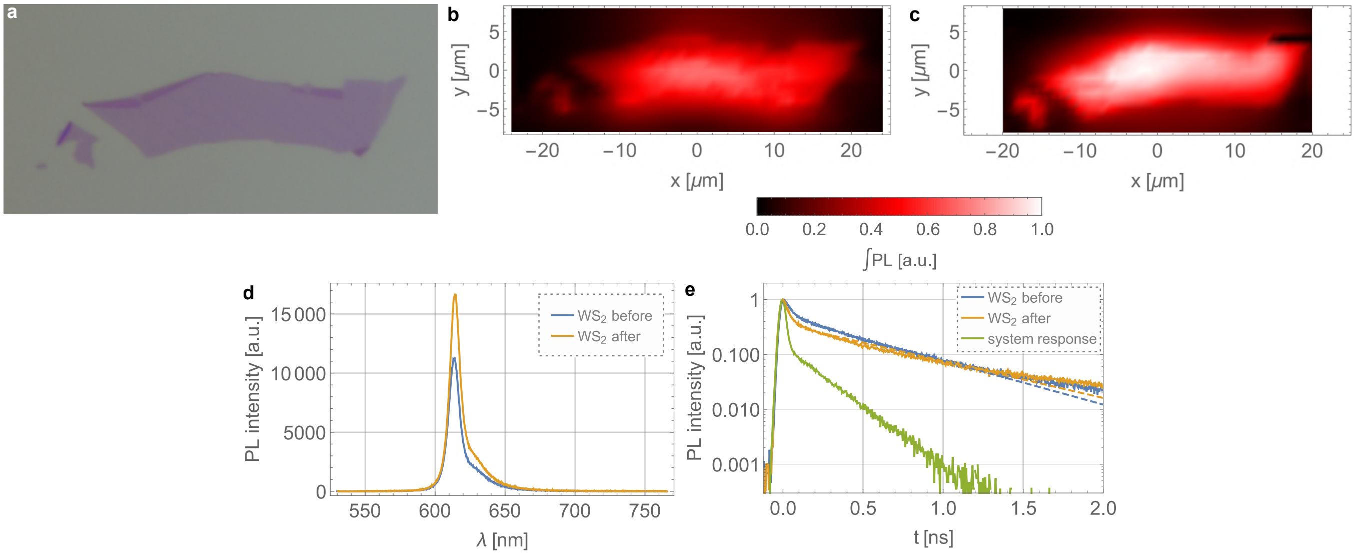

Supplementary Figure 5: Additional data set WS_2 after $F_\gamma = 5.68 \times 10^9\,\mathrm{cm^{-2}sr^{-1}MeV^{-1}}$. **a** Microscope image under 500× magnification. **b, c** Confocal PL map before and after irradiation. **d** The spectrum shows a PL increase of $\eta_{\mathrm{PL}} = 1.44$. **e** Carrier lifetime is increased from 542(4) to 647(6) ps ($\eta_\tau = 1.19$).

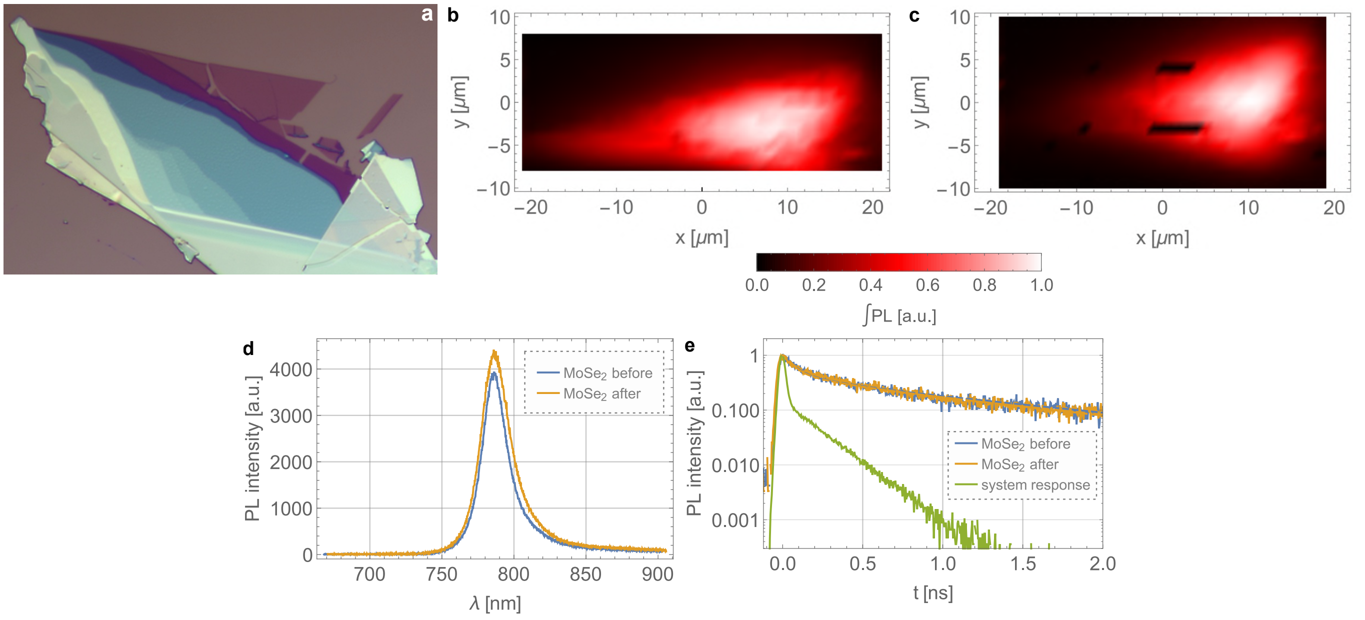

Supplementary Figure 6: Additional data set $MoSe_2$ after $F_\gamma = 10.89 \times 10^9\,\mathrm{cm^{-2}sr^{-1}MeV^{-1}}$. **a** Microscope image under 500× magnification. **b, c** Confocal PL map before and after irradiation. **d** The spectrum shows a PL increase of $\eta_{\mathrm{PL}} = 1.12$. **e** Carrier lifetime is decreased from 1472(61) to 1329(45) ps ($\eta_\tau = 0.90$).

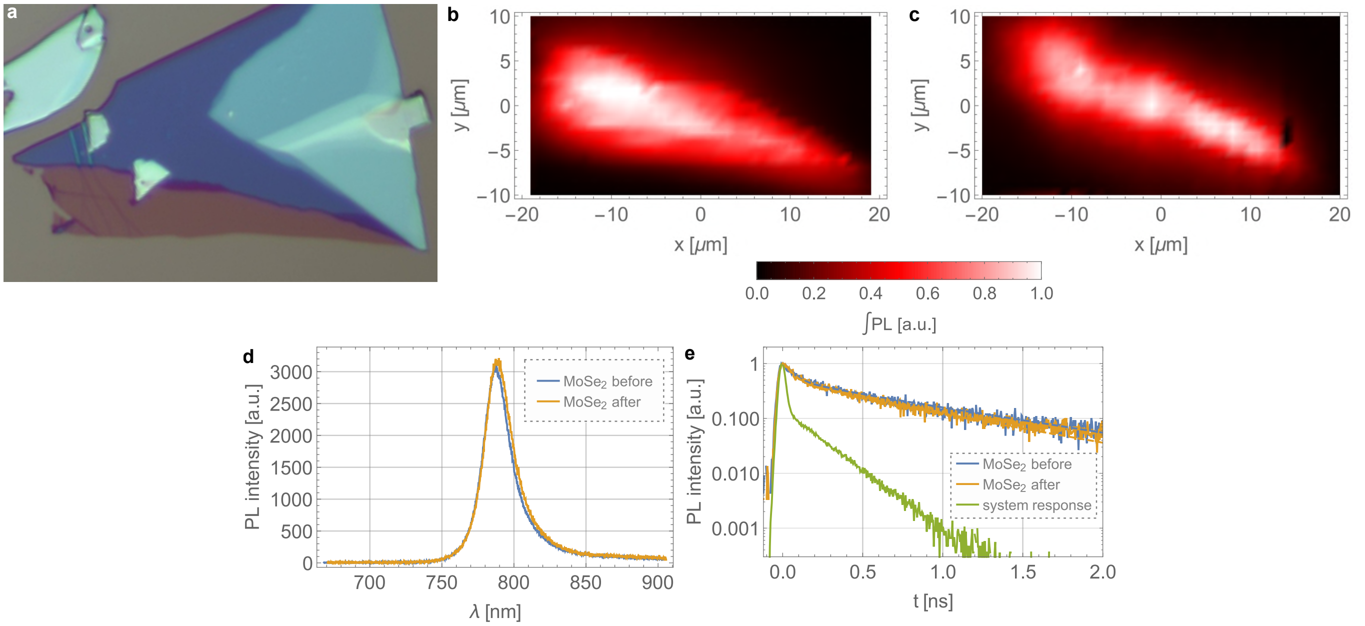

Supplementary Figure 7: Additional data set $MoSe_2$ after $F_\gamma = 1.15 \times 10^9\,\mathrm{cm^{-2}sr^{-1}MeV^{-1}}$. **a** Microscope image under 500× magnification. **b, c** Confocal PL map before and after irradiation. **d** The spectrum shows a PL increase of $\eta_{\mathrm{PL}} = 1.03$. **e** Carrier lifetime is decreased from 950(28) to 843(20) ps ($\eta_\tau = 0.89$).

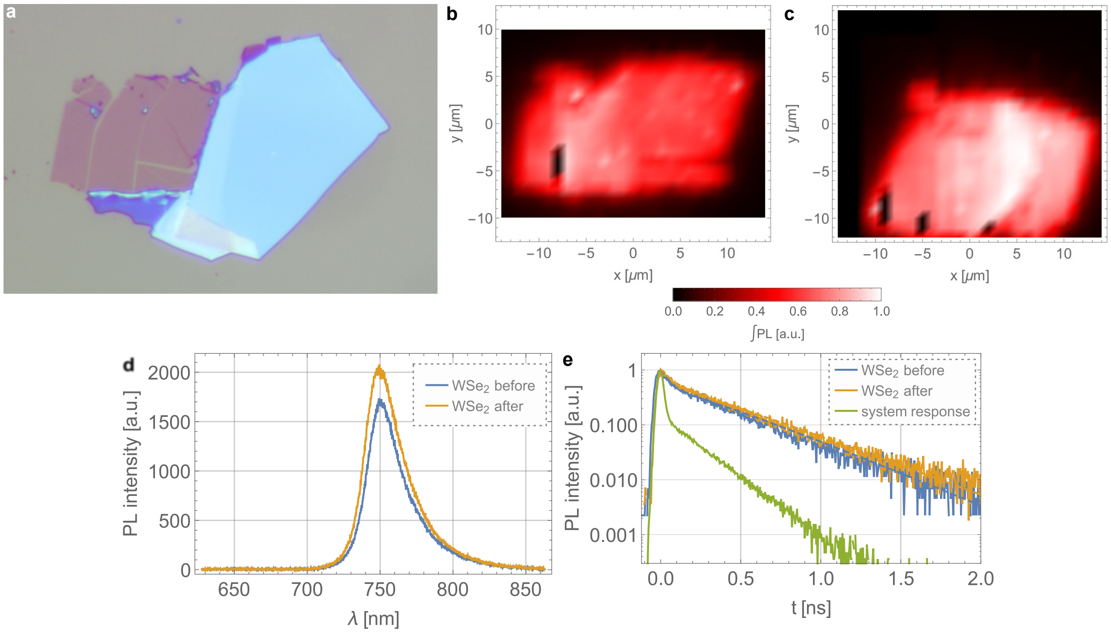

Supplementary Figure 8: Additional data set WSe_2 after $F_\gamma = 18.41 \times 10^9\,\mathrm{cm}^{-2}\mathrm{sr}^{-1}\mathrm{MeV}^{-1}$. **a** Microscope image under 500× magnification. **b, c** Confocal PL map before and after irradiation. **d** The spectrum shows a PL increase of $\eta_{\mathrm{PL}} = 1.25$. **e** Carrier lifetime is increased from 364(4) to 403(4) ps ($\eta_\tau = 1.11$).

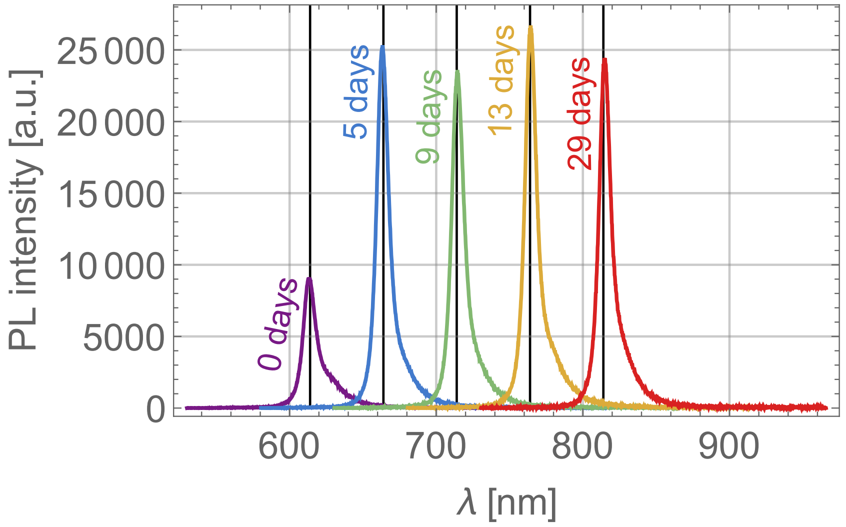

Supplementary Figure 9: Long-term stability of PL increase. The PL spectrum of the sample in Supplementary Figure 4 measured at different days. The irradiation took place at day 2. For clarity each subsequent spectra is shifted by 50 nm. The peak wavelength remained invariant (mean at 613.98 nm, visualized with black guidelines).

Supplementary Note 3: Proposed defect structures

The relaxed geometry obtained from our DFT calculations (see main text) showed that the length of the W-S bond is 2.42 Å for a pristine cell. The presence of a defect will result in relaxation of the atomic positions around the defect. Our calculations found that atoms nearest to the V_S defect move such that the nearest W-S bonds in the defect plane are 2.39 Å; the other nearby W-S bonds are 2.41 Å. In the case of the oxygen defect, the oxygen relaxes towards the transition metal, the length of the W-O bond is 2.07 Å while the other W-S bonds are roughly unchanged (~ 2.42 Å).

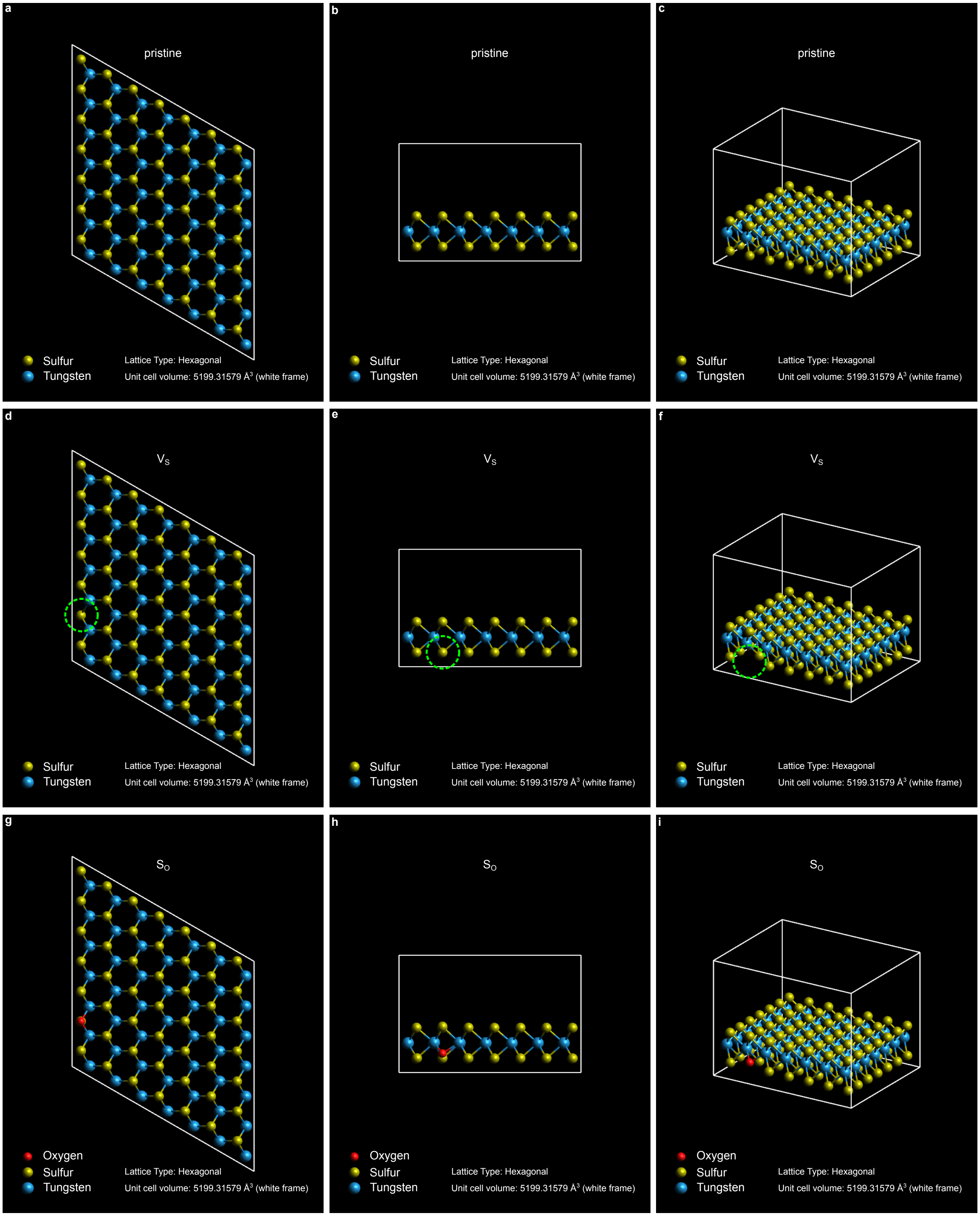

Supplementary Figure 10: Proposed defect structures in a 7×7 supercell WS_2. Pristine crystal, viewed along the Miller indices **a** $h, k, l = 0, 0, 0$; **b** $h, k, l = 0, -1, 0$; **c** $h, k, l = 1, -2, 1$. V_S defect, viewed along the Miller indices **d** $h, k, l = 0, 0, 0$; **e** $h, k, l = 0, -1, 0$; **f** $h, k, l = 1, -2, 1$. The green circle marks the position of the vacancy. S_O defect, viewed along the Miller indices **g** $h, k, l = 0, 0, 0$; **h** $h, k, l = 0, -1, 0$; **i** $h, k, l = 1, -2, 1$. For visibility, the defects shown here are at the edges of the supercell (this does not change the DFT calculations).

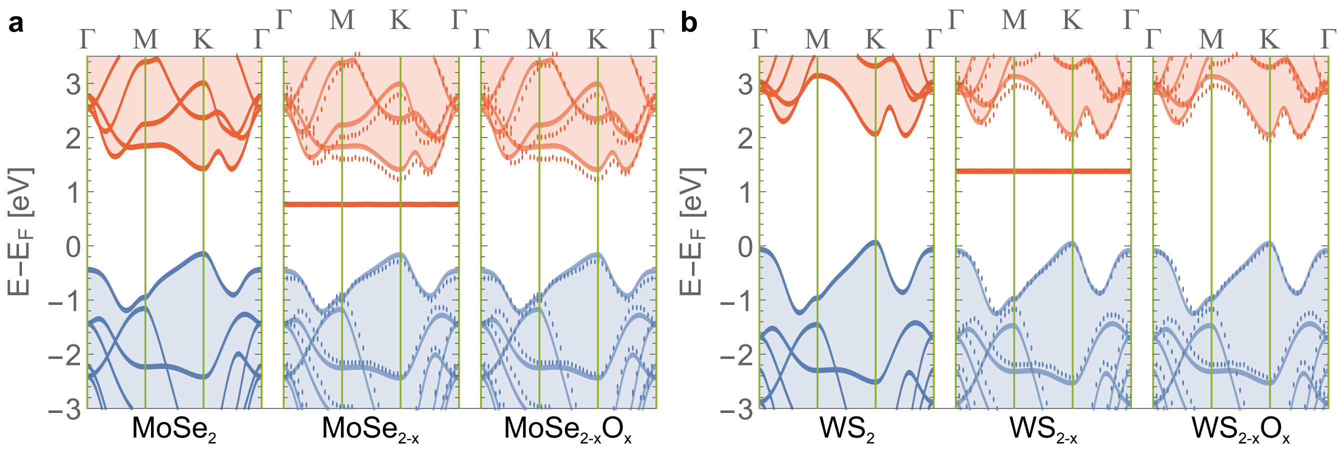

Supplementary Figure 11: DFT calculations. **a** DFT calculations of the bandstructure of pristine $MoSe_2$ (left), $MoSe_{2-x}$ (middle), $MoSe_{2-x}O_x$ (right) show that unlike the V_{Se} defect, the Se_O defect has no unoccupied deep mid-bandgap state. The middle and right bandstructure show the conduction and valence band from the primitive pristine unit cell (solid lines) overlayed with the conduction and valence band from the supercell calculations (dotted lines). **b** For a direct comparison we include the bandstructure of WS_2 here as well (see also main text). As the DFT calculations show, the defect state of the V_{Se} is closer to the conduction band ($\Delta E = 0.45\,\mathrm{eV}$ compared to $0.56\,\mathrm{eV}$ for the V_S defect). This means that the non-radiative charge capture cross section (CCS) of the V_S defect is smaller, as more phonons are required for the capture. For radiative charge capture this effect is reversed. With the energy difference of $0.45\,\mathrm{eV}$ to the conduction band, the V_{Se} defect has a smaller radiative CCS. The overall capture probability is given by defect density times capture cross section, so even though the non-radiative CCS for the V_{Se} defect is higher, with the much-reduced defect density the overall capture probability is lower.

Supplementary Note 4: Interactions of charge carriers with matter

The higher energies of the annual fluence spectra calculated with SPENVIS (see main text) are not directly accessible with our particle accelerators. To assess the damage mechanism at lower energies we calculate the stopping power for protons and electrons in 2D materials. The software used are SRIM[1] for protons and ESTAR[2] for electrons. For protons the damage mechanism is dominated by nuclear energy loss via Rutherford scattering. However, with a very low collision probability, even high-energy collisions produce only local point-like defects. Electron excitation in this energy regime generally does not produce defects. At extreme electronic energy losses electron-phonon coupling can lead to local heating. This could modify the material, but on the for space relevant energy scale this does not occur (only for highly relativistic protons). Electrons are excited, but then simply relax. Ionization does only play a minor, if any role.

The nuclear stopping power (see Supplementary Figure 12a) decreases with proton energy. The stopping power of electrons (see Supplementary Figure 12b) shows a similar trend at the for space relevant energies. At low energies the stopping power is dominated by collisions and as the collision cross section decreases with electron energy, the stopping power decreases as well. However, at higher energies, due to bremsstahlung the radiative stopping power dominates. Thus, both protons and electrons with higher energies cause less damage (at least on the relevant energy scales), and the fluence must be scaled accordingly. Unfortunately, as mentioned in the main text, the minimal fluence is already above what is expected, so no further scaling down is possible. In terms of space qualification this is not an issue, as the crystals get certified for even higher radiation doses.

We note that SRIM and ESTAR have been developed for bulk materials. In the relevant energy range the radiation damage is created by collisions between the impacting particle and the atoms in the 2D material. SRIM and ESTAR can handle this reasonably well. However, the programs use various approximations, including the assumption of a mean free path between scattering events. This approximation is not valid for monolayered 2D materials. Moreover, appropriate simulations carried out by Lehtinen et al.[3,4] show, that the type of defects in 2D differs from their bulk counterparts. Complex defects are formed due to the recoil of atoms in-plane. Furthermore, the simulations show that defect production probabilities decrease with increasing energies in the MeV range. So while one has to be careful with the absolute values for the stopping power of the 2D materials (see Supplementary Figure 12), SRIM and ESTAR nevertheless reproduce the qualitative trend of a decreasing stopping power with increasing particle energy

for the relevant energy range correctly.

In addition to the stopping power of the charge carriers in 2D materials, we also provide Monte Carlo simulations of the interaction of the protons and electrons with the Al shielding material of the spacecraft. The SPENVIS calculations assume a shielding thickness of 1.853 mm Al. Supplementary Figure 13 show trajectories of the electrons and protons with varying energy through such shield, generated with Monte Carlo methods using CASINO[5] and TRIM[1], respectively. Furthermore, Supplementary Figure 14 shows the maximal range in Al of the corresponding particles as a function of energy, which determines the shielding thickness.

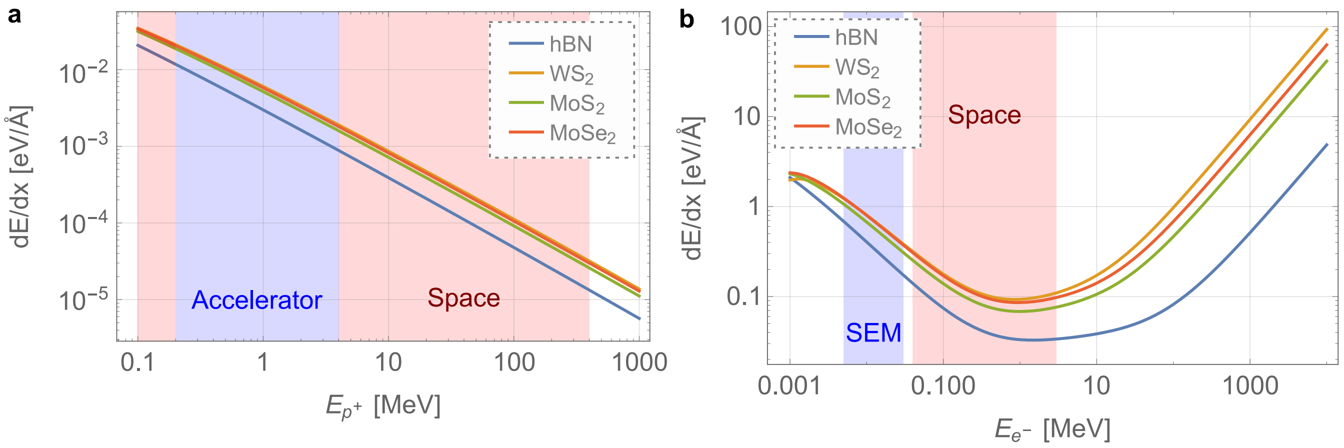

Supplementary Figure 12: Stopping power. The blue shaded areas indicate the energy range of the proton accelerator/SEM and the red shaded areas the expected proton energy range in space. **a** Nuclear stopping power for protons in various 2D materials. **b** Total stopping power for electrons in various 2D materials. At low energies, the stopping power is dominated by collisions, while at high energies it is dominated by radiation.

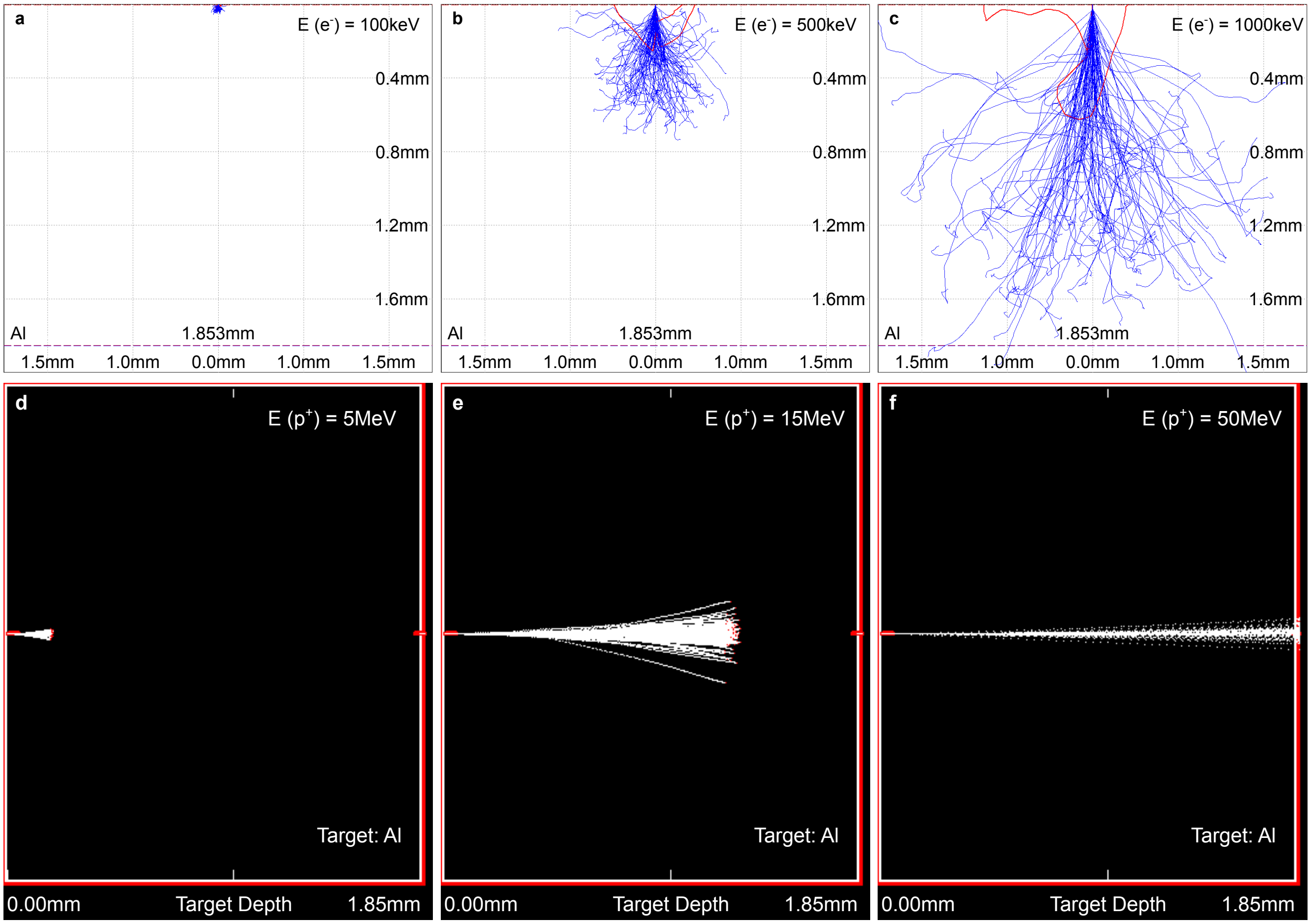

Supplementary Figure 13: Monte Carlo simulations. Electron trajectories in Al with energies **a** 100 keV, **b** 500 keV, and **c** 1 MeV, simulated with CASINO. Proton trajectories in Al with energies **d** 5 MeV, **e** 15 MeV, and **f** 50 MeV simulated with TRIM.

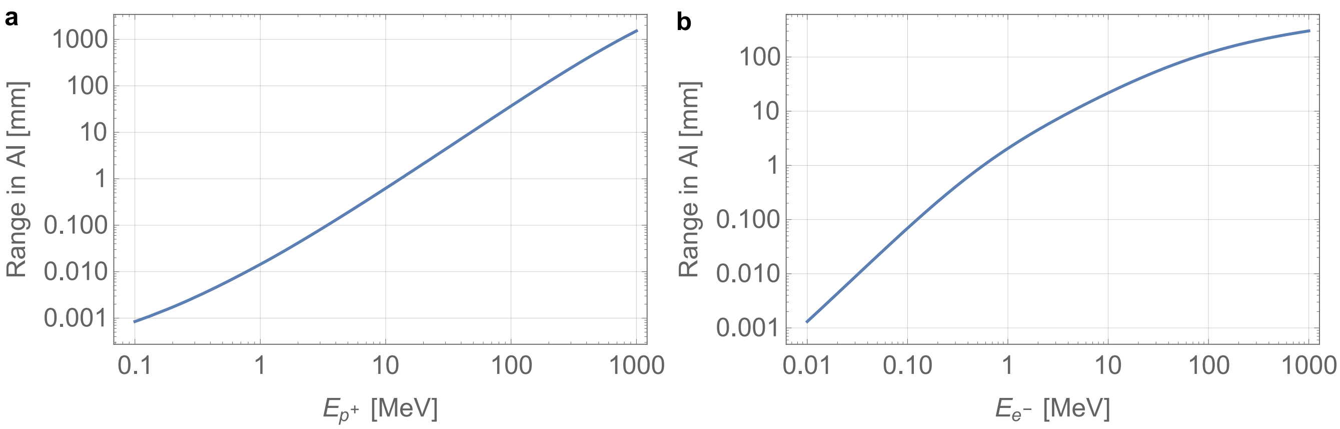

Supplementary Figure 14: Range of particles in Al. Projected range of **a** protons and **b** electrons in Al.

Supplementary Note 5: Proton irradiation

As stated in the main text, proton irradiation had no effect on any of the 2D materials at the tested fluences and energies. This was still true even after increasing the proton fluence 100-fold, to $10^{12}\,\mathrm{cm}^{-2}$. At 500 km altitude and an orbital inclination of 51.6° (which is the orbit with the highest flux) this fluence corresponds to 1386 years in orbit. Hence, we conclude that proton irradiation is no concern for 2D materials and devices in low Earth orbit (LEO). The in the following presented results (see Supplementary Figures 14 through 16) are exemplary for the full data set. We chose to show the same devices as in the main text, meaning that all samples in this section have been previously also irradiated with γ-rays. We note that we also added fresh samples to study both, isolated and combined radiation effects, but we saw no difference between these.

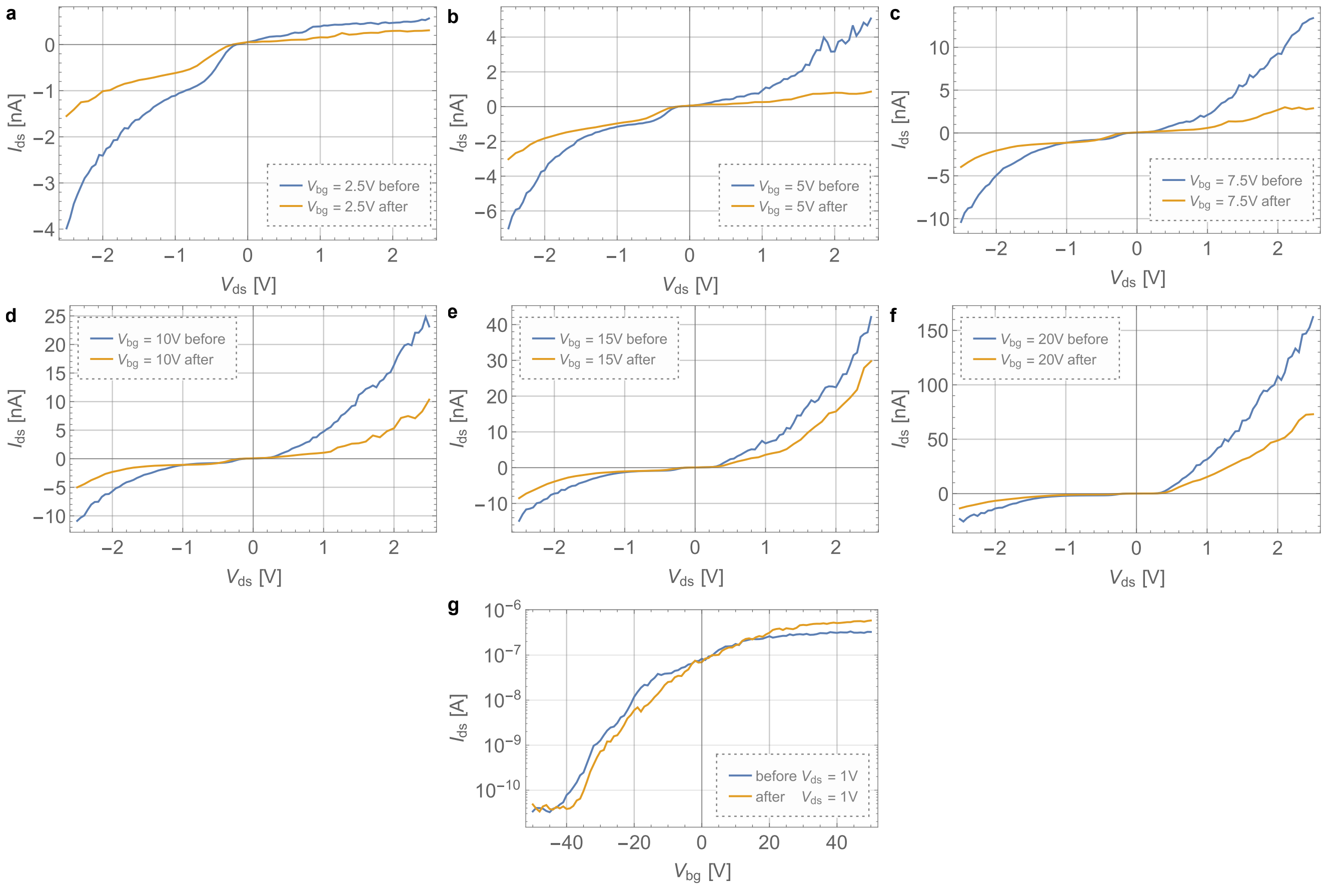

Supplementary Figure 15: Complete characterization of MoS_2 FET before / after p^+ irradiation. This sample has been previously irradiated with γ-rays (see Supplementary Figure 2). The fluence was $10^{10}\,\mathrm{cm}^{-2}$ at a proton energy of 2.5 MeV. **a-f** *I-V* curves at different back gate voltages. **g** The back gate sweep at a drain-source bias of 1 V shows no degradation in performance with the ON/OFF ratio increased from 10319 to 17479. The variations in *I-V* characteristics before and after the proton test are most likely due to temporal variations and are not actually caused by the radiation. This was confirmed by time-dependent measurements and caused by surface adsorption (see main text). In addition, *I-V* characteristics in general are highly dependent on the Schottky or contact resistance which varies across different measurements.

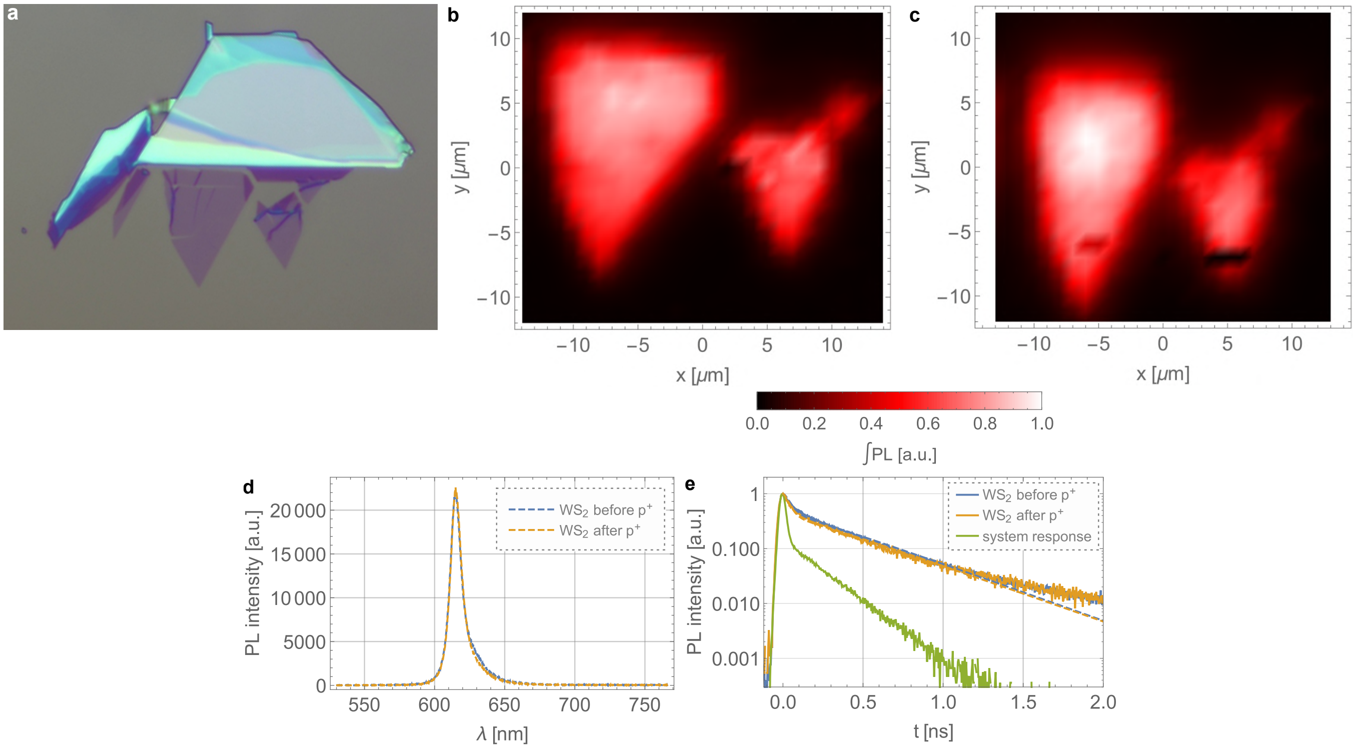

Supplementary Figure 16: WS_2 monolayer before / after irradiation with 2.5 MeV protons and a fluence $10^{10}\,cm^{-2}$. This sample has been previously irradiated with γ-rays (see Supplementary Figure 4). **a** Microscope image under 500× magnification. **b, c** Confocal PL map before and after irradiation. **d** The spectrum remained invariant with $\eta_{PL} = 0.96$. The exciton and trion peak positions were stable (before 614.85(2) nm and after 614.74(2) nm for excitons and before 620.99(15) and after 619.76 nm for trions), as well were the linewidths. **e** Carrier lifetime shows no change with 417(3) ps before and 425(2) ps after the proton irradiation ($\eta_\tau = 1.02$).

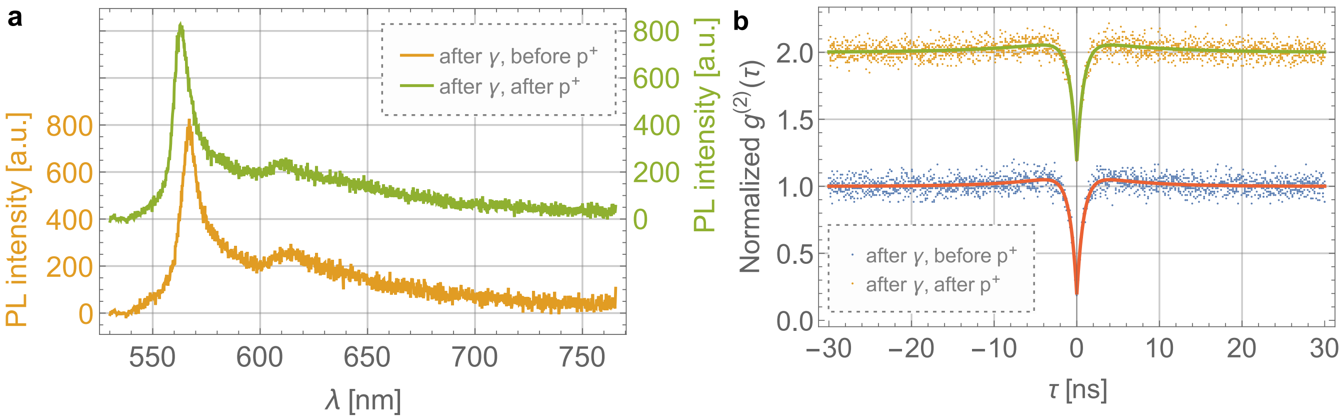

Supplementary Figure 17: Characterization of a quantum emitter in hBN before / after irradiation with 2.5 MeV protons and a fluence $10^{10}\,cm^{-2}$. This sample has been previously irradiated with γ-rays (see main text Figure 3). **a** The PL spectra (vertically offset for clarity) show no changes. **b** Second-order correlation function dipping at zero time delay to 0.188(25) before and to 0.185(23) after the irradiation. The values were obtained from fitting a three-level system (for clarity the fits are differently colored).

Supplementary Note 6: Electron irradiation

As stated in the main text, electron irradiation was able to cause significant damage on the optical properties of the 2D transition metal dichalcogenides (TMDs). A scanning electron microscope (SEM) was used as an electron accelerator (see main text Methods). During the first irradiation tests at 5 kV accelerating voltage and an electron fluence of 10^{13} cm^{-2}, we saw a strong decrease in PL, combined with a shortening in carrier lifetime (see Supplementary Figure 18). When changing the accelerating voltage to 30 kV and keeping the fluence constant we saw this effect weaken with increased electron energy (see Supplementary Figure 19). By pushing the SEM to its minimal fluence limit of 10^{10}cm^{-2}, we saw a further reduction of this effect (see Supplementary Figure 20), even at 5 kV accelerating voltage. Note that this is still three orders of magnitude above LEO radiation levels. We also extrapolate the damage effect on TMDs to be negligible in environments comparable to LEO.

The single-photon emitter in hBN were not affected by the electron irradiation. However, at locations at which the SEM was aligned and the electron beam focused (see Supplementary Figure 21), we saw a strong increase in emitter density. The electron fluence at these positions was up to 10^{18}cm^{-2}. The fact that intense electron irradiation can catalyze the formation of quantum emitters in hBN has been reported previously[6,7].

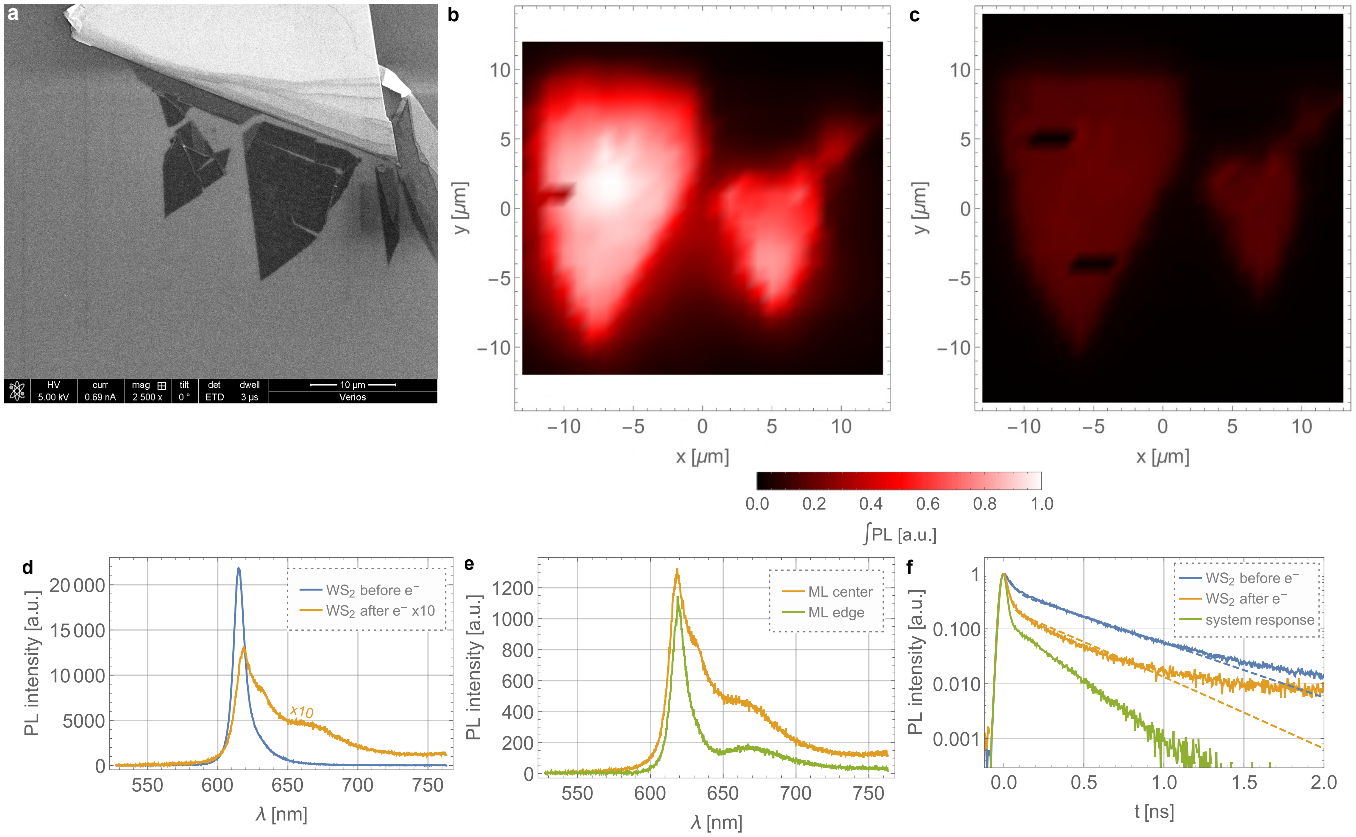

Supplementary Figure 18: WS_2 monolayer before / after irradiation with 5 keV electrons and a fluence of 10^{13} cm^{-2}. This sample has been previously irradiated with γ-rays (see Supplementary Figure 4) and protons (see Supplementary Figure 16. **a** SEM image under 2500× magnification. **b, c** Confocal PL map before and after irradiation. **d** The PL emission was strongly decreased as well as its shaped changed significantly. **e** Unlike for the center of the monolayer (ML), at the edges of the ML, the spectral shape was more comparable to the un-irradiated averaged spectrum. **f** The carrier lifetime was decreased from 433(2) ps before to 328(5) ps after the electron irradiation.

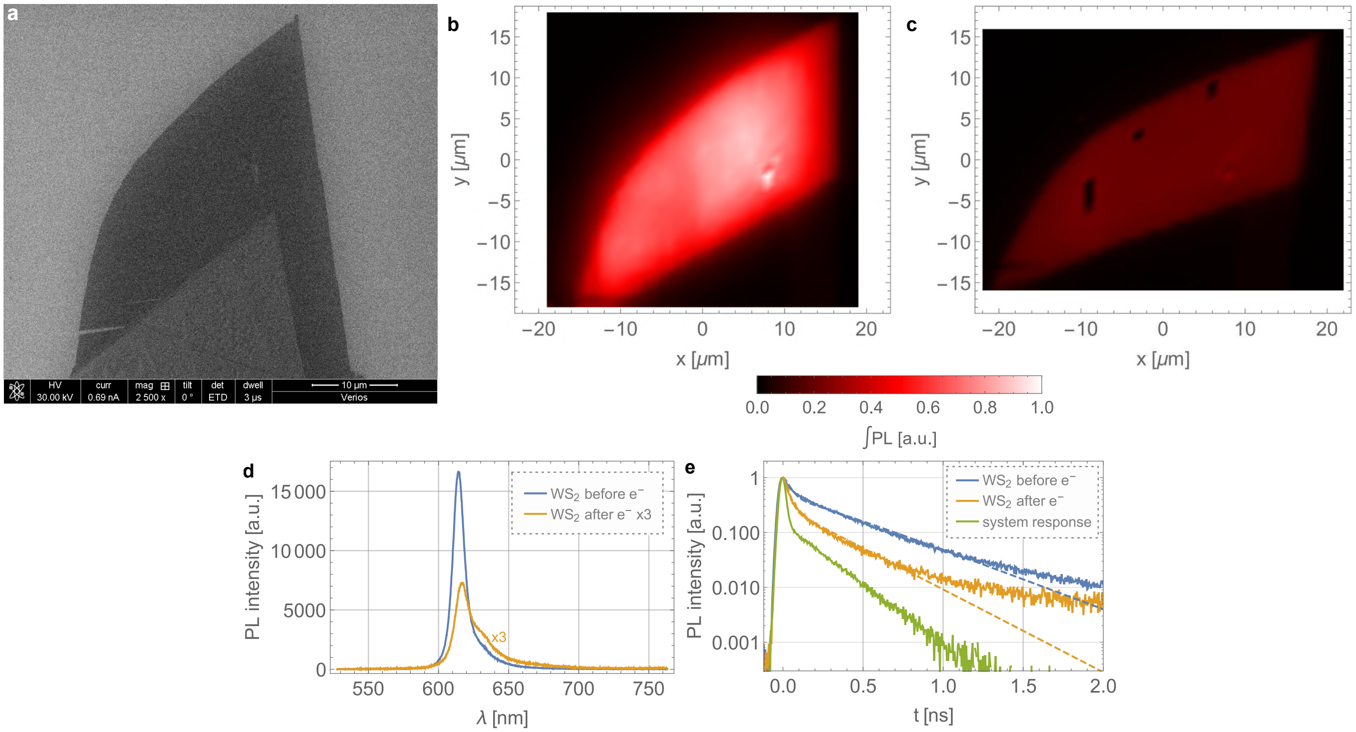

Supplementary Figure 19: WS_2 monolayer before / after irradiation with 30 keV electrons and a fluence of $10^{13}\,\mathrm{cm}^{-2}$. This sample has been previously irradiated with γ-rays and protons. **a** SEM image under 2500× magnification. **b, c** Confocal PL map before and after irradiation. **d** The PL emission was decreased as well as its shaped changed (both change less than with 5 keV electrons). **e** The carrier lifetime was decreased from 395(2) ps before to 287(4) ps after the electron irradiation.

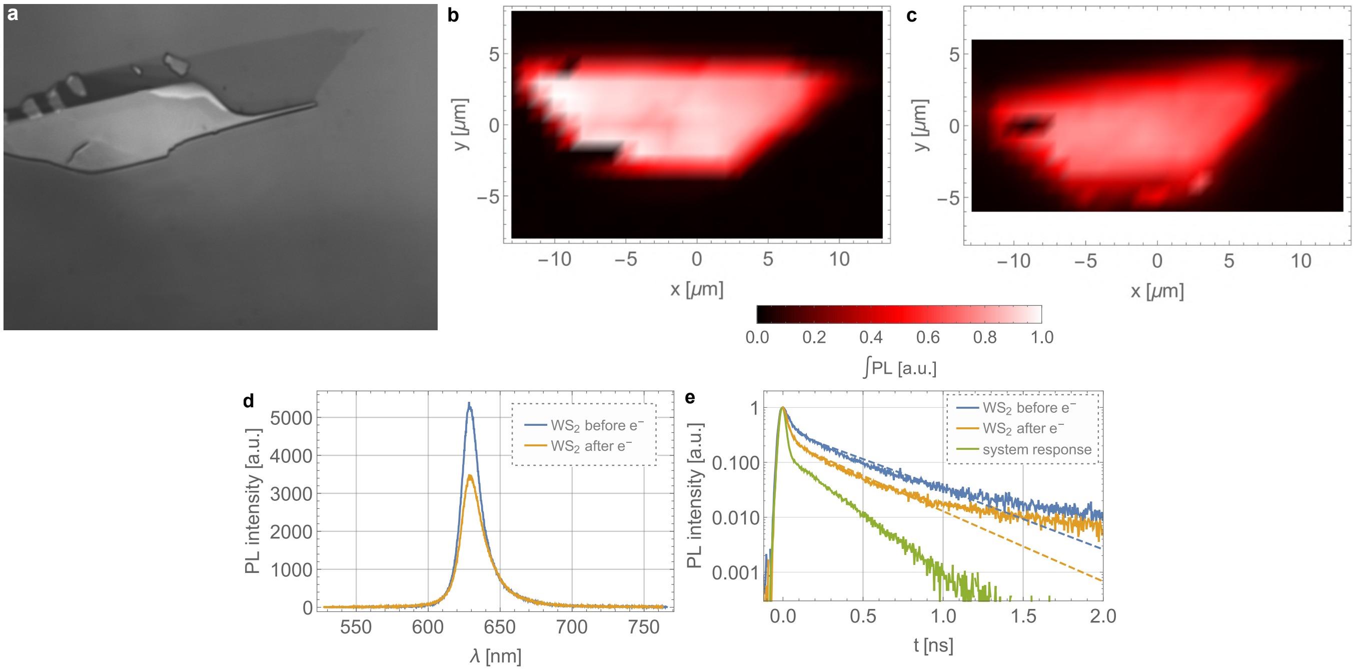

Supplementary Figure 20: WS_2 monolayer before / after irradiation with 5 keV electrons and a fluence of $10^{10}\,\mathrm{cm}^{-2}$. This sample has been previously irradiated with γ-rays and protons. **a** Microscope image under 1000× magnification. **b, c** Confocal PL map before and after irradiation. **d** The PL emission was decreased only marginally (compared to the tests with a higher fluence), despite the lower electron energy which causes the larger damage. **e** The carrier lifetime was only decreased from 391(5) ps before to 314(5) ps after the electron irradiation.

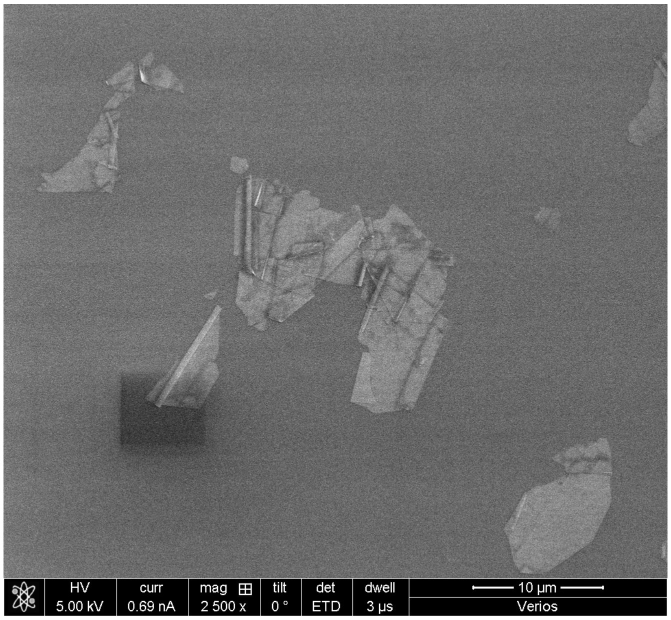

Supplementary Figure 21: SEM image of an hBN crystal. The SEM image shows a dark area at which the electron beam was aligned and focused. The electrons make the surface reactive and carbon-contaminations caused by residual organic materials in the SEM apparatus itself are bonded at areas with intense electron irradiation. At these areas the number of quantum emitters is strongly increased.

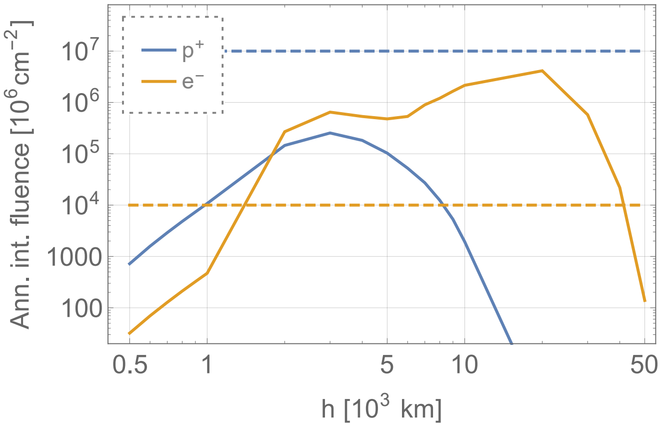

Supplementary Figure 22: Integrated annual particle fluence. The annual fluence spectra after 1.85 mm of Al shielding are integrated over the full energy range. The proton fluence always remains nearly two orders of magnitude below the damage onset threshold (dashed lines correspondingly colored). The electron fluence exceeds the observed damage onset threshold at altitudes > 1000 km. The shielding explains the leap in electron fluence at 2000 km: The electron energy increases with altitude and thus actually trapped electrons can penetrate the shielding. At lower energies the electrons originate from secondary processes of the protons interacting with the shielding material. By using an appropriate shield (5.8 mm graded Al/Ta with a Ta to Al mass ratio of 35%), the electron fluence can be kept below the damage threshold.

Supplementary References

[1] Ziegler, J. F., Ziegler, M. & Biersack, J. SRIM - The stopping and range of ions in matter. *Nucl. Instr. Meth. Phys. Res. B* **268**, 1818 – 1823 (2010).

[2] Berger, M., Coursey, J., Zucker, M. & Chang, J. ESTAR, PSTAR, and ASTAR: Computer programs for calculating stopping-power and range tables for electrons, protons, and helium ions (2005). http://physics.nist.gov/Star, National Institute of Standards and Technology, Gaithersburg, MD.

[3] Lehtinen, O. et al. Effects of ion bombardment on a two-dimensional target: Atomistic simulations of graphene irradiation. *Phys. Rev. B* **81**, 153401 (2010).

[4] Lehtinen, O. et al. Production of defects in hexagonal boron nitride monolayer under ion irradiation. *Nucl. Instrum. Methods Phys. Res. B* **269**, 1327 – 1331 (2011).

[5] Drouin, D. et al. CASINO V2.42 - A Fast and Easy-to-use Modeling Tool for Scanning Electron Microscopy and Microanalysis Users. *Scanning* **29**, 92–101 (2007).

[6] Choi, S. et al. Engineering and localization of quantum emitters in large hexagonal boron nitride layers. *ACS Appl. Mater. Interfaces* **8**, 29642–29648 (2016).

[7] Ngoc My Duong, H. et al. Effects of high-energy electron irradiation on quantum emitters in hexagonal boron nitride. *ACS Appl. Mater. Interfaces* **10**, 24886–24891 (2018).
